# Short- and Long-Term Efficacy of Intensive Rehabilitation Treatment on Balance and Gait in Parkinsonian Patients: A Preliminary Study with a 1-Year Followup

**DOI:** 10.1155/2013/583278

**Published:** 2013-05-26

**Authors:** Giuseppe Frazzitta, Gabriella Bertotti, Davide Uccellini, Natalia Boveri, R. Rovescala, Gianni Pezzoli, Roberto Maestri

**Affiliations:** ^1^Department of Parkinson Disease Rehabilitation, “Moriggia-Pelascini” Hospital, Via Pelascini 3, 22015 Gravedona ed Uniti (CO), Italy; ^2^Fondazione Europea Ricerca Biomedica FERB, “S.Isidoro” Hospital, Via Ospedale 34, 24069 Trescore Balneario, Italy; ^3^Department of Neurorehabilitation, Scientific Institute of Montescano, S. Maugeri Foundation IRCCS, Via per Montescano 31, 27040 Montescano, Italy; ^4^Department of Neurology, Tradate Hospital, Piazzale Zaniboni 1, 21049 Tradate, Italy; ^5^Parkinson Institute, Istituti Clinici di Perfezionamento, Via Bignami 3, 20100 Milano, Italy; ^6^Department of Biomedical Engineering, Scientific Institute of Montescano, S. Maugeri Foundation IRCCS, Via per Montescano 31, 27040 Montescano, Italy

## Abstract

Parkinson's disease (PD) is a neurodegenerative disease in which gait and balance disturbances are relevant symptoms that respond poorly to pharmacological treatment. The aim of this study was to investigate whether a 4-week inpatient multidisciplinary intensive rehabilitation treatment (MIRT) is effective in improving balance and gait and whether improvements persist at a one-year followup. We studied 20 PD inpatients (stage 3 Hoehn-Yahr) who underwent a MIRT. Outcome measures were UPDRS items for balance (30), falls (13), and walk (29), Berg Balance Scale, six-minute walking test, Timed Up and Go Test, and Comfortable-Fast gait speeds. Patients were evaluated at admission, at the end of the 4-week treatment, and at a 1-year followup. Pharmacological therapy was unchanged during MIRT and follow-up. All outcome measures improved significantly at the end of treatment. At 1-year follow-up control, UPDRS walk and Comfortable-Fast gait speeds still maintained better values with respect to admission (*P* = 0.009, *P* = 0.03, and *P* = 0.02, resp.), while the remaining scales did not differ significantly. Our results demonstrate that the MIRT was effective in improving balance and gait and that the improvement in gait performances was partially maintained also after 1 year.

## 1. Introduction

Parkinson's disease (PD) is a neurodegenerative disorder characterized by different motor symptoms (rigidity, akinesia, tremor, and impairment of balance and gait). Even though pharmacological treatment has changed the natural course of disease, gait and balance worsen over time and these disturbances progressively lead to major disability [1, 2].

Postural instability leads to falls: PD subjects often fall because they respond to a sudden loss of balance with abnormally short steps that are inadequate to recover equilibrium [3, 4].

Moreover, Parkinsonian patients present continuous gait disturbances (shorter stride length, reduced gait speed, increased variability of stride, and increased double support time) that, often associated with freezing and festination, lead to frequent falls [5, 6].

In the last five years, several studies have shown that physiotherapy strategies, including cueing techniques, treadmill training, and cognitive movement strategies, are useful in improving balance and gait in PD patients [7–10].

However, whether the effects of the rehabilitation on gait and balance persist over time and what the optimal content of exercise intervention is (the kind of exercises, the intensity, and the duration of treatment) remain open questions [11]. 

It has been recently hypothesized that the best approach to rehabilitation in Parkinson disease is a multidisciplinary treatment [12].

There are very few multidisciplinary approaches in rehabilitation of Parkinson disease [13, 14]. 

Gait and balance disorders have a multifactorial aetiology and a multidisciplinary approach which might be more effective for the different components responsible for these symptoms.

In this context, we developed a 4-week inpatient multidisciplinary intensive rehabilitation treatment (MIRT) in which standard physical therapy techniques were associated with treadmill with auditory and visual cues (treadmill plus) and a stabilometric platform. 

The present study was devised: (a) to test whether our protocol is effective in improving balance and gait during supervised multidisciplinary treatment; and (b) to verify whether improvements persist after one year of unsupervised treatment.

## 2. Methods 

We enrolled 20 patients (7 males and 13 females) (Table 1) with a diagnosis of “clinically probable” idiopathic PD according to Gelb et al. [15], in Hoehn-Yahr stage 3, with the ability to walk without any assistance, who had fallen in the last year at least 2 times, with mini-mental state examination score ≥26, without any relevant comorbidity or vestibular/visual dysfunctions limiting locomotion or balance, and who had been assuming stable dopaminergic therapy in the last 4 weeks. patients were screened by a neurologist specialized in movement disorders, and eligible patients were admitted to the Rehabilitation Institute, for a 4-week rehabilitation treatment. The study was approved by the local Scientific Committee and Institutional Review Board (Fondazione S. Maugeri, IRCCS, Istituto Scientifico di Montescano). Written informed consent was obtained from all patients before participation.

The outcome measures were the Unified Parkinson's Disease Rating Scale (UPDRS) [16] items for balance and falls (UPDRS_30 and UPDRS_13, resp.) and the Berg Balance Scale (BBS) [17] for balance as well as the UPDRS items for walk (UPDRS_29), the six-minute walking test (6MWT) (the participant walked along a straight path), the Timed Up and Go Test (TUG), and the Comfortable (CGS) and Fast gait speeds (FGS) (10 meters straight path) for the gait [18, 19].

Patients were evaluated at the beginning and at the end of 4-week inpatient rehabilitation treatment and at a 1-year followup in the morning, one hour after the first dose of Levodopa, by a neurologist specialized in movement disorders, who was blinded to the study design. 

### 2.1. Intervention

Patients were admitted to the Rehabilitation Institute where they underwent a MIRT: a 4-week cycle of physiotherapy that entailed three daily sessions (two in the morning and one in the afternoon, one hour each), 5 days a week. The MIRT involved different professionals: physiatrists, neurologists, physiotherapists, occupational therapists, nurses, psychologists, and nutritionists. The rehabilitation treatment was administered each day in different sessions. The first session comprised cardiovascular warm-up activities, relaxation exercises, muscle-stretching (scapular, hip flexor, hamstring, and gastrocnemius muscles), exercises to improve the range of motion of spinal, pelvic and scapular joints, and exercises to improve the functionality of abdominal muscles and postural changes in the supine position. The second session included exercises to improve balance and gait using a stabilometric platform with a visual cue and treadmill training associated with auditory and visual cues (treadmill plus). In brief, using the stabilometric platform, the patients were asked to follow a circular pathway on the screen by means of a cursor sensitive to the movements made by their feet on the platform. For treadmill training, the visual cue was a target, displayed on a screen, which the patient had to reach within a stride while the auditory cue consisted of musical beats, synchronized with the visual cues, with a frequency of 0.5 c/s [10]. All treadmill treatments were performed in an aerobic manner with a heart rate reserve ≤60% and a maximum speed of treadmill scrolling of 3.5 Km/h.

The last was an occupational therapy session with the aim of improving autonomy in daily living activities: transfers from sitting to standing, rolling from supine to sitting and from sitting to supine, dressing, use of tools, and exercises to improve hand functionality and skills (e.g., using screws and bolts). At the end of MIRT, the patients were instructed to continue a set of learned exercises in order to maintain functionality of the spine, scapular, and pelvic joints All patients received the same exercise program, that was part of the overall inpatient program. Patients were instructed to perform the exercises every day and to walk at least 30 minutes a day. The adherence to the exercise program and walking was not measured over this 11-month unsupervised period. 

### 2.2. Sample Size Computation

Published studies report a standard error of measurement (SEM) equal to 30 m, 1.8, 0.59 s, 0.06 s, and 0.09 s for 6MWT, BBS, TUG, CGS, and FGS, respectively [20, 21]. We expected an effect size around 35 m, 4, 1.2 s, 0.12 s, and 0.15 s for the same variables. Hence, to detect a change with a two-tailed type I error of 0.05 and a power of 80%, the estimated sample size (the largest among all estimates) was 14 patients. We chose a conservative sample size of 20 patients.

### 2.3. Statistical Analysis

Descriptive statistics are given as mean ± SD. The Shapiro-Wilk statistic was used to test the normality of the distribution of all variables. 

To investigate the primary and secondary end point, the time course of each functional variable considered was assessed by repeated measurements analysis of variance with three repeated measurements: admission, discharge, and 1-year followup. Pairwise comparisons (discharge versus admission and 1-year followup versus admission) were carried out by contrast analysis in repeated measurements analysis of variance. A *P*-value <0.05 was considered statistically significant. All analyses were carried out using the SAS/STAT statistical package, release 9.2 (SAS Institute Inc., Cary, NC, U.S.A.).

## 3. Results

All patients completed the MIRT (all patients attended the three daily sessions included in the program), and there were no dropouts at the end of study. Table 1 reports demographical characteristics of studied patients. During the MIRT and followup pharmacological therapy was unchanged. The functional characteristics of patients at admission, discharge, and at the 1-year followup are reported in Table 2. The *P* values for the comparison with the measurement performed at admission, taken as the control level against which successive measurements are compared by contrast analysis, are also reported.

All considered scales improved significantly at the end of the rehabilitation treatment (*P* < 0.0001 for UPDRS_29, UPDRS_30, 6MWT, BBS and TUG, *P* = 0.012, *P* = 0.0002, and *P* = 0.0007 for UPDRS_13, CGS, and FGS, resp.). 

At the 1-year followup, UPDRS_29, CGS, and FGS still maintained better values with respect to admission (*P* = 0.009, *P* = 0.03, and *P* = 0.02, resp.), while the values of the remaining scales did not differ significantly. 

## 4. Discussion

This study was aimed primarily to test the efficacy of MIRT on balance and gait and to investigate whether the beneficial effects persist after one year of unsupervised treatment. Our results demonstrate that the treatment was very effective in improving postural stability and gait during the inpatient treatment. 

At the 1-year followup, most of the scales related to gait evaluation (UPDRS 29, CGS, and FGS) were still significantly improved with respect to the values observed at enrollment, and the other functional variables exhibited values close to those observed at admission. None of the patients had to increase the drug dosage during the followup and it is possible to exclude a pharmacological intervention on patients' outcome.

These results point out that, despite the degenerative nature of their illness, Parkinsonian patients could improve balance and gait during a multidisciplinary inpatient intensive treatment and partially maintain the result over time if they continue to make regular physical activity. It is well known that the natural history of Parkinson's disease leads to a progressive impairment of gait and balance and to a progressive increase in Levodopa dosage which is scarcely effective in improving these symptoms and brings about several side effects, such as dyskinesia and hallucinations [2, 22–24].

Moreover, balance and gait may not be related to dysfunctions of the dopamine systems [25–27].

These considerations push to develop new strategies for the treatment of these symptoms. 

There is great interest in using exercise for gait and balance disorders, as exercise may have both symptomatic effects as well as disease-modifying effects [28]. 

Gait and balance disturbances are complex phenomena with multifactorial problems involving vestibular, proprioceptive, and musculoskeletal dysfunctions. Hence, a multidisciplinary approach, delivered by an efficient and well coordinated multidisciplinary team, is likely to be more effective for the different components responsible for these symptoms. Indeed, our multidisciplinary protocol was not designed as simply the sum of a set of interventions delivered to the patient independently, but each intervention was integrated into a streamlined team approach, aimed to the development of a tailored rehabilitation program. 

Treadmill training is effective in reducing the variability of gait, enhancing gait speed, and reducing freezing of gait in PD, and several balance training programs have been developed by different groups which also demonstrated short-term efficacy [29–32]. 

It has also been suggested that the intensity of treatment is a key factor in achieving better results [31–34]. We hypothesize that the persistence of improved gait performance at the 1-year followup might be related to the peculiarity of our rehabilitation strategy, which was developed taking into account all these aspects.

As far as the functional variables related to balance are concerned, one year after treatment their values were virtually the same as at first admission. We think this is an important result which can be better appreciated considering the chronic-degenerative nature of PD. 

## 5. Study Limitation

The lack of a control group and the lack of a control over the execution and adherence of the exercises during followup are the most important limitations of the study. However based on the lack of drop-outs and the good motor performance of the patients at followup, we can hypothesize a good adherence to instructions given at discharge. This hypothesis is further supported by the fact that none of the patients had to increase the drug dosage during the study.

## 6. Conclusion

Our findings indicate that balance and gait disturbances can be effectively countered by a multidisciplinary intensive inpatient treatment. The promotion of physical activity should be considered a valid option to maintain a good motor performance and autonomy in daily activity and to delay the increase in drug dosage with related adverse effects, although this needs to be tested in future well-controlled trials. 

## Figures and Tables

**Table 1:** Demographic data.

Age	Sex (M/F)	Years of disease	Hoehn-Yahr	UPDRS tot	UPDRS III	UPDRS II
71 ± 7	7/13	8.3 ± 2.1	3	34.74	18.18	14.48

**Table 2:** Functional characteristics of studied patients at admission, discharge, and at the 1-year followup.

	*N*	Admission	Discharge	*P*	1-year followup	*P*
UPDRS_13	20	0.7 ± 0.9	0.1 ± 0.4	0.012	0.7 ± 0.9	0.012
UPDRS_30	20	1.8 ± 0.5	1.1 ± 0.4	<0.0001	1.4 ± 0.6	0.77
BBS	20	45.1 ± 7.4	50.8 ± 6.8	<0.0001	45.4 ± 7.5	0.86
UPDRS_29	20	1.9 ± 0.9	1.1 ± 0.6	<0.0001	1.6 ± 0.9	0.009
sixMWT (m)	20	258.1 ± 86.1	322.7 ± 107.4	<0.0001	271.1 ± 82.5	0.098
TUG (s)	20	12.4 ± 3.0	9.6 ± 2.5	<0.0001	11.6 ± 2.8	0.31
CGS (m/s)	20	0.87 ± 0.18	1.00 ± 0.13	0.0002	0.96 ± 0.18	0.03
FGS (m/s)	20	1.07 ± 0.22	1.24 ± 0.19	0.0007	1.17 ± 0.20	0.02

## References

[1] Franzén E, Paquette C, Gurfinkel VS, Cordo PJ, Nutt JG, Horak FB (2009). Reduced performance in balance, walking and turning tasks is associated with increased neck tone in Parkinson’s disease. *Experimental Neurology*.

[2] Horak FB, Frank J, Nutt J (1996). Effects of dopamine on postural control in parkinsonian subjects: scaling, set, and tone. *Journal of Neurophysiology*.

[3] Bloem BR, Grimbergen YAM, Cramer M, Willemsen M, Zwinderman AH (2001). Prospective assessment of falls in Parkinson’s disease. *Journal of Neurology*.

[4] Wood BH, Bilclough JA, Bowron A, Walker RW (2002). Incidence and prediction of falls in Parkinson’s disease: a prospective multidisciplinary study. *Journal of Neurology, Neurosurgery and Psychiatry*.

[5] Baltadjieva R, Giladi N, Gruendlinger L, Peretz C, Hausdorff JM (2006). Marked alterations in the gait timing and rhythmicity of patients with de novo Parkinson’s disease. *European Journal of Neuroscience*.

[6] Bloem BR, Hausdorff JM, Visser JE, Giladi N (2004). Falls and freezing of Gait in Parkinson’s disease: a review of two interconnected, episodic phenomena. *Movement Disorders*.

[7] Keus SHJ, Munneke M, Nijkrake MJ, Kwakkel G, Bloem BR (2009). Physical therapy in Parkinson’s disease: evolution and future challenges. *Movement Disorders*.

[8] Lim I, van Wegen E, Jones D (2010). Does cueing training improve physical activity in patients with Parkinson’s disease?. *Neurorehabilitation and Neural Repair*.

[9] Morris ME, Iansek R, Kirkwood B (2009). A randomized controlled trial of movement strategies compared with exercise for people with Parkinson’s disease. *Movement Disorders*.

[10] Frazzitta G, Maestri R, Uccellini D, Bertotti G, Abelli P (2009). Rehabilitation treatment of gait in patients with Parkinson’s disease with freezing: a comparison between two physical therapy protocols using visual and auditory cues with or without treadmill training. *Movement Disorders*.

[11] Tomlinson CL, Patel S, Meek C (2012). Physiotherapy versus placebo or no intervention in Parkinson's disease. *Cochrane Database of Systematic Reviews*.

[12] Post B, van der Eijk M, Munneke M, Bloem BR (2011). Multidisciplinary care for Parkinson’s disease: not if, but how!. *Practical Neurology*.

[13] Johnston M, Chu E (2010). Does attendance at a multidisciplinary outpatient rehabilitation program for people with parkinson’s disease produce quantitative short term or long term improvements? A systematic review. *NeuroRehabilitation*.

[14] Ellis T, Katz DI, White DK, DePiero TJ, Hohler AD, Saint-Hilaire M (2008). Effectiveness of an inpatient multidisciplinary rehabilitation program for people with Parkinson disease. *Physical Therapy*.

[15] Gelb DJ, Oliver E, Gilman S (1999). Diagnostic criteria for Parkinson’s disease. *Archives of Neurology*.

[16] Fahn S, Elton RL, Fahn S, Marsden CD, Calne D, Goldstein M (1987). Unified Parkinson’s disease rating scale. *Recent Developments in Parkinson’s Disease*.

[17] Berg KO, Wood-Dauphinee SL, Williams JI, Maki B (1992). Measuring balance in the elderly: validation of an instrument. *Canadian Journal of Public Health*.

[18] Steffen TM, Hacker TA, Mollinger L (2002). Age- and gender-related test performance in community-dwelling elderly people: six-minute walk test, berg balance scale, timed up & go test, and gait speeds. *Physical Therapy*.

[19] Morris S, Morris ME, Iansek R (2001). Reliability of measurements obtained with the timed “up & go” test in people with Parkinson’s disease. *Physical Therapy*.

[20] Steffen T, Seney M (2008). Test-retest reliability and minimal detectable change on balance and ambulation tests, the 36-item short-form health survey, and the unified Parkinson disease rating scale in people with parkinsonism. *Physical Therapy*.

[21] Lim LIIK, van Wegen EEH, de Goede CJT (2005). Measuring gait and gait-related activities in Parkinson’s patients own home environment: a reliability, responsiveness and feasibility study. *Parkinsonism and Related Disorders*.

[22] Burleigh-Jacobs A, Horak FB, Nutt JG, Obeso JA (1997). Step initiation in Parkinson’s disease: influence of levodopa and external sensory triggers. *Movement Disorders*.

[23] Gottwald MD, Aminoff MJ (2011). Therapies for dopaminergic-induced dyskinesias in Parkinson disease. *Annals of Neurology*.

[24] Friedman JH (2010). Parkinson’s disease psychosis 2010: a review article. *Parkinsonism and Related Disorders*.

[25] Bonnet AM, Loria Y, Saint-Hilaire MH, Lhermitte F, Agid Y (1987). Does long-term aggravation of Parkinson’s disease result from nondopaminergic lesions?. *Neurology*.

[26] Dietz V, Berger W, Horstmann GA (1988). Posture in Parkinson’s disease: impairment of reflexes and programming. *Annals of Neurology*.

[27] Jacobs JV, Horak FB (2006). Abnormal proprioceptive-motor integration contributes to hypometric postural responses of subjects with parkinson’s disease. *Neuroscience*.

[28] Voss MW, Prakash RS, Erickson KI (2010). Plasticity of brain networks in a randomized intervention trial of exercise training in older adults. *Frontiers in Aging Neuroscience*.

[29] Frenkel-Toledo S, Giladi N, Peretz C, Herman T, Gruendlinger L, Hausdorff JM (2005). Treadmill walking as an external pacemaker to improve gait rhythm and stability in Parkinson’s disease. *Movement Disorders*.

[30] Mehrholz J, Friis R, Kugler J, Twork S, Storch A, Pohl M (2010). Treadmill training for patients with Parkinson’s disease. *Cochrane Database of Systematic Reviews*.

[31] Gobbi LTB, Oliveira-Ferreira MDT, Caetano MJD (2009). Exercise programs improve mobility and balance in people with Parkinson’s disease. *Parkinsonism and Related Disorders*.

[32] Smania N, Corato E, Tinazzi M (2010). Effect of balance training on postural instability in patients with idiopathic parkinsong’s disease. *Neurorehabilitation and Neural Repair*.

[33] Ebersbach G, Ebersbach A, Edler D (2010). Comparing exercise in Parkinson’s disease—the Berlin LSVT BIG study. *Movement Disorders*.

[34] Shulman LM, Katzel LI, Frederick IV (2013). Randomized clinical trial of 3 types of physical exercise for patients with Parkinson disease. *JAMA Neurology*.

